# Thrust Symmetry Engineering in Lithographically Defined Bubble-Propelled Micromotors via Multi-Orifice Exhaust-Port Design

**DOI:** 10.1021/acsomega.6c05214

**Published:** 2026-08-01

**Authors:** Isao Shitanda, Takuma Suzuki, Kentaro Zama, Yoshinao Hoshi, Masanori Hayase, Masayuki Itagaki

**Affiliations:** † Department of Pure and Applied Chemistry, Faculty of Science and Technology, 13258 Tokyo University of Science, 2641, Yamazaki, Noda, Chiba 278-8510, Japan; ‡ Research Institute for Science and Technology, Tokyo University of Science, 2641 Yamazaki, Noda, Chiba 278-8510, Japan

## Abstract

Bubble-driven catalytic micromotors generate thrust through gas–liquid interfacial processes at the inner surfaces of the catalyst; however, systematic control over the thrust-vector symmetry through the geometric design of the exhaust port layout has not been demonstrated. Here, we report a lithographic fabrication platform that produces stacked Au/Pt hollow micromotors with independently programmable catalytic exhaust-port configurations—single, two, and three orifices—which enable a direct experimental investigation of the extent to which the interface-defined port geometry strongly influences the propulsion behavior. Using a single-orifice trapezoidal micromotor as the baseline system, we establish concentration-dependent propulsion at 73 μm s^–1^ (5 wt % H_2_O_2_) and 636 μm s^–1^ (15 wt % H_2_O_2_). Extending the platform to a two-orifice design reveals persistent curved trajectories (∼450 μm s^–1^, 15 wt % H_2_O_2_), indicating that even small geometric asymmetries in exhaust-port placement can produce measurable and persistent torque. A three-orifice design incorporating a central forward-thrust port and two lateral steering ports enables the straight-line motion to be recovered under uniform fuel conditions, consistent with improved thrust-vector balance for the mirror-symmetric port placement. In a nonuniform fuel environment imposed by a H_2_O_2_ gel source, the three-orifice micromotor exhibits directional motion toward the low-concentration region, consistent with differential bubble generation across the lateral ports. These results establish that, within the present platform and under the same material and catalytic conditions, the number, placement, and symmetry of exhaust ports strongly affect the observed propulsion state, providing an interface-programmable geometric route to activate micromotor behavior without changing the material composition or fuel chemistry.

## Graphical abstract

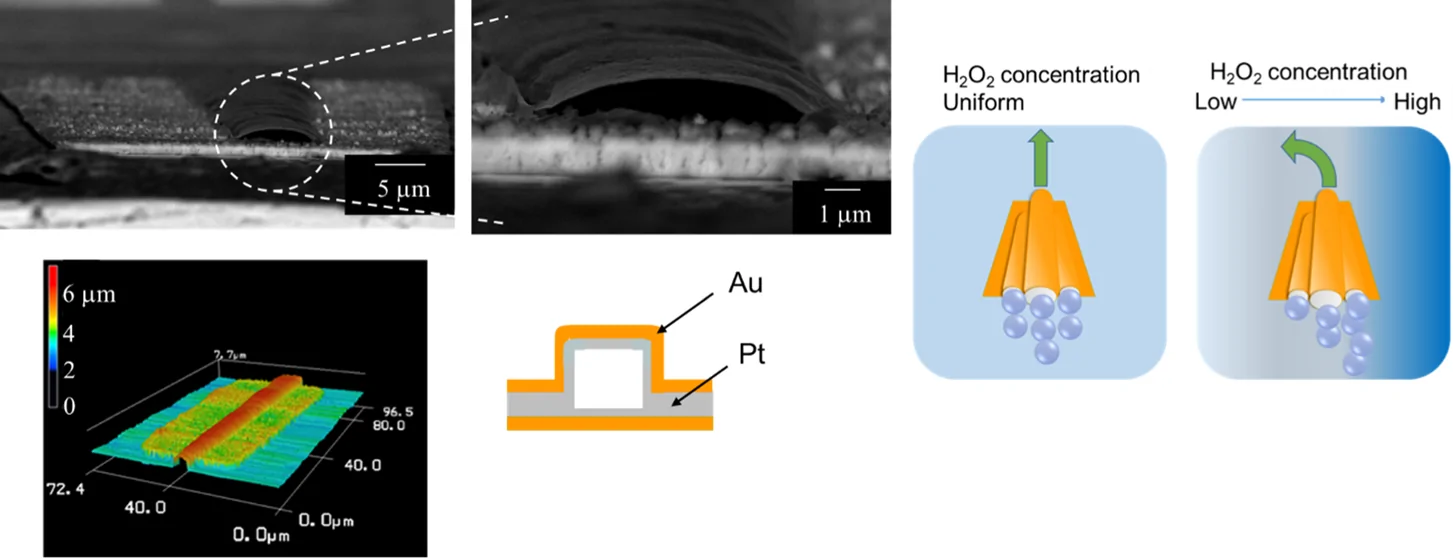

## 1 Introduction

Autonomous micro- and nanomotors that convert chemical energy into directed mechanical motion in fluid environments have attracted substantial interest as functional microsystems for mixing, cargo transport, environmental remediation, and sensing.^1–4^ Among the diverse propulsion mechanisms that have been explored, bubble-driven catalytic propulsion—in which the catalytic decomposition of H_2_O_2_ at the Pt surface results in the directional ejection of O_2_ bubbles at the gas–liquid interface to produce thrust—has become prominent owing to its characteristically high speeds that often exceed several hundred micrometers per second. In these systems, propulsion is fundamentally an interfacial process; the catalytic inner surface defines the site of gas evolution, and the exhaust port geometry shapes the way in which the resulting gas–liquid momentum transfer is converted into a macroscopic thrust vector.^5,6^

Bubble propulsion in catalytic micromotors is not governed by a single process: capillarity, bubble growth, and bubble expulsion can act individually or simultaneously, and the dominant contribution can vary with the surface tension, fuel concentration, and viscosity.^7^ In the present hollow architecture, the O_2_ generated by H_2_O_2_ decomposition at the inner Pt surface grows and is ejected through the exhaust ports; however, bubble-growth dynamics and capillary effects at the gas–liquid interface may also contribute to the local force generation near each port. We therefore describe the resulting motion as a combined gas–liquid interfacial process—gas generation, bubble growth, bubble detachment and ejection, and interfacial capillary effects—rather than as bubble expulsion alone. In the present study, this coupled sequence is not resolved as separate rate processes; instead, its spatial distribution across the exhaust ports is treated as the effective origin of local thrust generation, as developed in Sections 3.1, 3.4, and 3.5.

The two principal fabrication routes for bubble-driven micromotors have intrinsic limitations rooted in the way the process, material interfaces, and geometry are interrelated. The first of these routes, template-assisted electropolymerization through porous polycarbonate membranes, yields conical-tube micromotors with excellent propulsion performance,^5,8,9^ yet the shape of the tube is dictated by the profile of the membrane pore, and the outer-layer material is restricted to conducting polymers that are electropolymerizable.^8^ Although the other fabrication route, strain-driven roll-up of prestressed metallic nanomembranes, affords greater material flexibility,^10–13^ the achievable tube radius and semicone angle are governed by the residual-stress balance, which limits the design freedom.^14^ Both approaches have the disadvantage that the micromotor geometry—and therefore the architecture of the catalytic interface—is an output of the fabrication process rather than a freely chosen design input.

A further limitation of existing approaches is their predominant focus on single-orifice, axially symmetric tubular, or conical architectures, which led to the conclusion that the propulsion speed depends on channel geometry parameters such as the semicone angle, tube length, and opening diameters.^15–17^ This means that the question regarding the influence of the number and spatial arrangement of exhaust ports (the openings through which bubbles are ejected) on the resultant thrust vector and macroscopic motion state has received little experimental attention. Specifically, the effect of using single-, two-, and multiorifice exhaust port configurations fabricated under identical material, catalytic, and process conditions has not yet been systematically compared. This makes it difficult to isolate the role of the port geometry from confounding variables, such as differences in the catalytic loading, material composition, or fabrication method. In a single-orifice system, the thrust vector is determined primarily by the channel geometry and catalytic surface area. When multiple orifices are present, the net thrust becomes a vector sum of the individual port contributions; in the case of a port layout that is mirror-symmetric, the lateral components would cancel each other, and the micromotor would move in a straight line, whereas broken symmetry would produce a net torque and curved trajectories. This geometric degree-of-freedom multiorifice symmetry control has not yet been explored as a design variable for programming propulsion behavior. Few systems attempting to address this important issue have been reported,^18,19^ neither has a controlled comparison of single-, two-, or three-orifice configurations within a unified platform been forthcoming. Recent bubble-driven micromotors and microrobots have demonstrated a range of capabilities, including magnetically guided navigation, multimodal locomotion near interfaces, integrated assembly and micromanipulation, biosensing, and inertial mass sensing.^20–23^ These advances have been achieved mainly by introducing external fields, additional materials, or extra functional components. In contrast, the present work keeps the material composition, catalytic surface, and fuel chemistry fixed and treats the lithographically defined exhaust-port layout as the design variable, so that single-, two-, and three-orifice configurations can be compared on a common platform.

In this study, we address these oversights by developing a lithographic fabrication platform^12^ that produces stacked hollow micromotors with independently programmable exhaust-port layouts while maintaining identical Au/Pt structural and catalytic interface architectures across designs. We fabricate micromotors with three configurations: (i) a single-orifice design with a trapezoidal baseline, (ii) a two-orifice design to probe the sensitivity to the thrust asymmetry, and (iii) a three-orifice design that separates forward thrust from lateral steering and recovers straight-line motion by exploiting mirror symmetry. We further examined the motion response of the three-orifice system in a nonuniform fuel environment. The results establish a design principle—thrust symmetry engineering in which, within the present platform, the propulsion state is strongly influenced by the geometric layout of the catalytic exhaust ports.

## 2 Experimental Section

### 2.1 Preparation of Materials and Substrate

Copper plates (25 mm × 25 mm) with a vacuum-evaporated Ag sacrificial layer (RD-1300, Sunvac; ∼1 × 10^–3^ Pa; 150 A; ∼40 s) served as substrates. A positive-tone photoresist OFPR-800LB (Tokyo Ohka Kogyo) with an HMDS adhesion promoter (OAP; Tokyo Ohka Kogyo) was used for all the patterning steps. Au electrodeposition used Tempe Resist K-91H (Japan Pure Chemical; pH 7.3–7.8; 50 °C). Pt electrodeposition was performed using Platanicide PT-10 (Matex Japan; pH < 1.0; RT). Pt sputtering: Quick Coater SC-701HMCII (Sanyu Denshi). All fabrication conditions were identical across the three configurations, unless otherwise stated.

**Safety note.** Concentrated H_2_O_2_ is a strong oxidizer. Appropriate PPE was used.

### 2.2 Photolithographic Patterning

HMDS and OFPR-800LB were spin-coated (1000 rpm, 10 s; 3000 rpm, 30 s) and prebaked (100 °C, 5 min). The outer layer patterns were exposed (mask aligner K307-PS, Kyowa Riken; Hg lamp HB-25105AA, Ushio; 12 s), developed (NMD-3, 90 s), and postbaked (100 °C, 15 min). For the single-orifice micromotor, the outer-layer pattern was trapezoidal (35/40/80 μm). For the two- and three-orifice systems, modified photomask designs define the multiport geometries. Inner-layer patterning for the three-orifice system used μPG501 laser direct-write (Heidelberg Instruments; 35 ms, −1) for submicrometer overlay accuracy.

### 2.3 Au/Pt Electrodeposition

A three-electrode cell (Teflon Pt wire CE, sat. KCl Ag/AgCl DJ-RE, EC Frontier; potentiostat HA-501G, Hokuto Denko). Au: −5.4 × 10^–3^ A cm^–2^, 90 s, 50 °C. Pt: −1.9 × 10^–3^ A cm^–2^, 72 s, RT. Identical for all configurations.

### 2.4 Inner-Layer Patterning and Hollow-Structure Definition

The second HMDS/OFPR-800LB layer defined the sacrificial resist island(s) as single trapezoidal (1-orifice), V-shaped chevron (2-orifice), or three-pronged (3-orifice). After development (NMD-3, 90 s) and postbaking (100 °C, 15 min), the resist islands served as void templates.

### 2.5 Pt Sputtering and Secondary Au Deposition

Pt sputter: ∼100 nm (Ar, ∼2.0 Pa, 20 mA, 0.5 kV, 4 min 30 s). Secondary Au: −5.4 × 10^–3^ A cm^–2^, 90 s, 50 °C.

### 2.6 Release and Purification

Immersion in acetone (∼20 min) dissolved all resists. The Ag was dissolved in 35% HNO_3_. Finally, the structures were subjected to centrifugation washing (6000 rpm, 5 min, ×5) in ultrapure water.

### 2.7 Propulsion Evaluation

Optical microscopy was conducted on a VHX-200/100F instrument (VH-Z100, Keyence; 15 fps). Uniform experiments were carried out by placing the desired concentration of H_2_O_2_ on a glass slide. In nonuniform experiments, H_2_O_2_-agar gel was placed 2 cm from the observation field. The negative control consisted of Au-only particles of the same shape, without Pt. The speed was determined on the basis of a frame-by-frame analysis.

## 3 Results and Discussion

### 3.1 Concept and Fabrication Strategy

In bubble-driven catalytic micromotors, O_2_ gas evolves at the inner surface of a Pt catalyst. The micromotor is propelled by the subsequent directional ejection of these bubbles through the exhaust ports, as determined by the number, size, and spatial arrangement of these openings at the gas–liquid interface.^15,17^ For a single-orifice geometry, thrust is directed along the channel axis, and the micromotor moves in a straight line (Figure 1a). When two exhaust ports are introduced, the net propulsion becomes the vector sum of the two port-level thrusts. Any geometric asymmetry between the ports produces unequal bubble ejection, a net lateral force component, and a persistent torque that deflects the trajectory into a curve (Figure 1b). Introducing a third centrally placed forward-thrust port with two laterally placed ports positioned in mirror symmetry about the forward axis allows the lateral thrust components to cancel and restore rectilinear motion under uniform fuel conditions (Figure 1c). Exposure to a spatial fuel gradient causes the same mirror-symmetric three-orifice architecture to asymmetrically activate the two lateral ports, which produces a thrust imbalance that deflects the trajectory toward the low-concentration region, whereas the central port maintains forward propulsion (Figure 1d). Together, these four configurations establish a unified physical framework in which the propulsion states emerge from the geometric composition of the thrust vectors, thus defining the principle of thrust symmetry engineering. Within this framework, programmable transport is interpreted as the macroscopic consequence of port-resolved bubble events—gas generation, bubble growth, detachment and ejection, and the associated interfacial capillary effects—and the present study focuses experimentally on how the spatial distribution of these coupled processes across the exhaust ports translates into thrust imbalance or thrust cancellation, rather than resolving each as a separate rate process.

**Figure 1:**
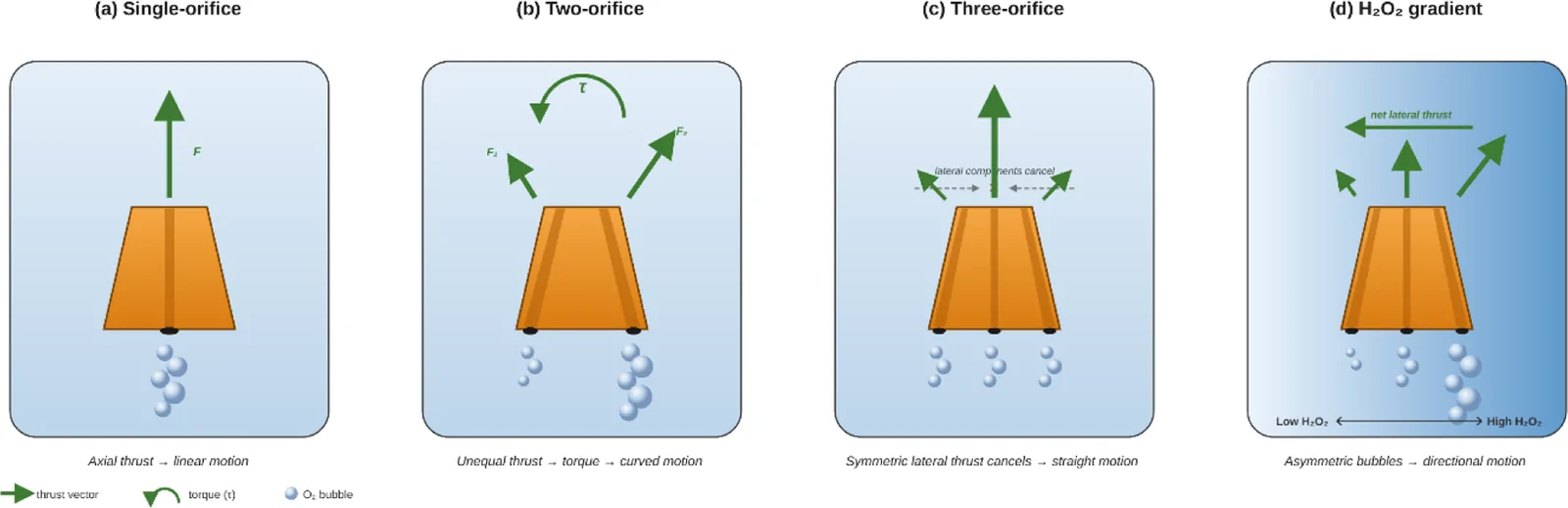
Conceptual framework of thrust symmetry engineering in bubble-propelled micromotors. (a) Single-orifice configuration: a single axial thrust vector for straight motion. (b) Two-orifice configuration: geometric asymmetry produces unequal thrust vectors for a net torque and curved motion. (c) Three-orifice configuration: restores straight motion through mirror-symmetric cancellation of lateral thrust components while maintaining forward propulsion. (d) Under a concentration gradient, differential activation of lateral ports produces a controlled thrust imbalance, resulting in directional motion toward the low-concentration region.

Realizing this design principle requires a fabrication route in which the exhaust port layout can be specified independent of the material composition, catalytic loading, and overall device footprint. The two commonly used fabrication routes, template-assisted electropolymerization^5,8,9^ and the strain-driven roll-up of prestressed nanomembranes^10–13^ both couple the final geometry to process-level constraints (the membrane pore profile and residual-stress balance), and therefore do not permit arbitrary multiorifice layouts to be compared on a common platform. Our solution to this problem was to develop a lithographically defined stacked hollow fabrication process that decouples the exhaust port geometry from the catalytic interface chemistry (Figure 2). The photoresist pattern of the outer layer defines the overall micromotor footprint, whereas a second, inner-layer photoresist island defines the internal void geometry from which the exhaust ports are ultimately derived. That is, sequential Au/Pt electrodeposition and Pt sputtering are employed to build the structural shell and catalytic inner surface around this resist template, with final resist dissolution releasing a hollow, multilayered microstructure with the designed exhaust-port layout.^12^ Because only the inner-layer photomask differs among the three configurations—single-, two-, and three-orifice—while all electrodeposition, sputtering, and release conditions remain identical, this platform preserves the material and catalytic interface identity across all the designs. This preservation enables direct experimental assessment of the thrust symmetry concept established in Figure 1.

**Figure 2:**
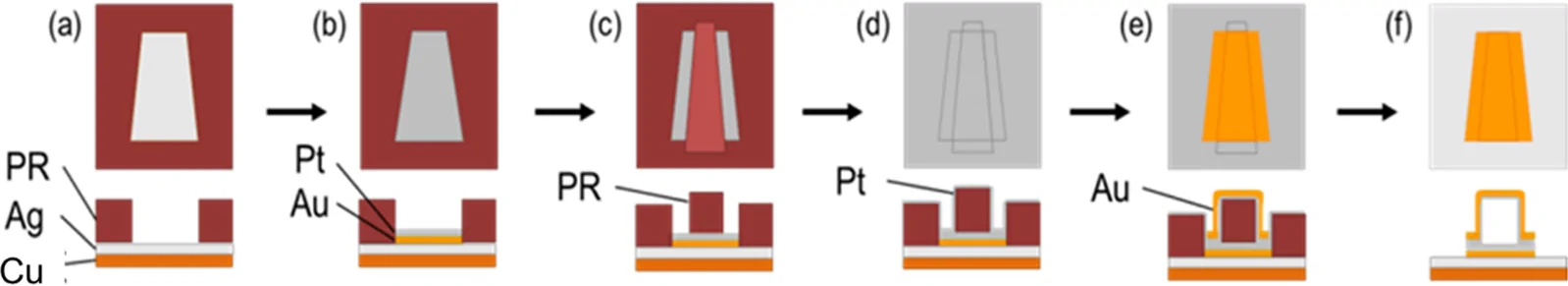
Fabrication process of lithographically defined multiorifice micromotors. (a) Patterning the outer photoresist (PR) layer onto the Cu/Ag substrate. (b) Electrodeposition of the outer Au/Pt structural layers. (c) Patterning of the inner photoresist layer that defines the internal void geometry. (d) Pt sputtering to form the catalytic inner surface. (e) Secondary Au electrodeposition to complete the multilayer shell. (f) Removal of the photoresist template and sacrificial layer to yield the released hollow micromotor with the designed exhaust-port configuration. Top-view (upper row) and cross-sectional (lower row) schematics are shown.

### 3.2 Fabrication Platform

Figure 2 summarizes the stepwise fabrication route, including outer-layer patterning, Au/Pt electrodeposition, inner-layer patterning, Pt sputtering, secondary Au deposition, and finally, release of the hollow micromotor.^12^ The photomask designs are detailed in Figure S1; the theoretical speed model guiding the geometry selection is presented in Figure S2.^16,17^ The electrochemical cell and deposition conditions are shown in Figures S3 and S4. The key parameters (Table 1) were held constant across all configurations. Optical microscopy confirmed the fidelity of the process at each stage (Figure S5). Cross-sectional FE-SEM and laser profilometry (Figure S6) confirmed a hollow cavity (∼1.33 μm height, ∼2.71 μm total stack). A variant with *D*_in_ ≈ *D*_out_ demonstrated no sustained propulsion (Figure S7 and Movie S3) as validation for the required geometric asymmetry.^17^

**Table 1:** Key Fabrication Conditions (Identical for All Configurations)

step	parameter	value
Au electrodeposition	current density/time	–5.4 × 10^–3^ A cm^–2^/90 s (50 °C)
Pt electrodeposition	current density/time	–1.9 × 10^–3^ A cm^–2^/72 s (RT)
Pt sputtering	conditions	20 mA, 0.5 kV, Ar ∼2.0 Pa, 4.5 min, ∼100 nm

The released single-orifice micromotors (Figure 3) had dimensions of 166 μm (length), 90 μm (top width), and 122 μm (base width) (Table 2). These dimensions exceed those of the designed photomask (80/35/40 μm) because of lateral metal overgrowth during electrodeposition and slight expansion upon release from the substrate. To document the geometric reproducibility of the lithographic process, the experimentally traceable geometric and motion-analysis information available from the archived data for each configuration is summarized in Table S1.

**Figure 3:**
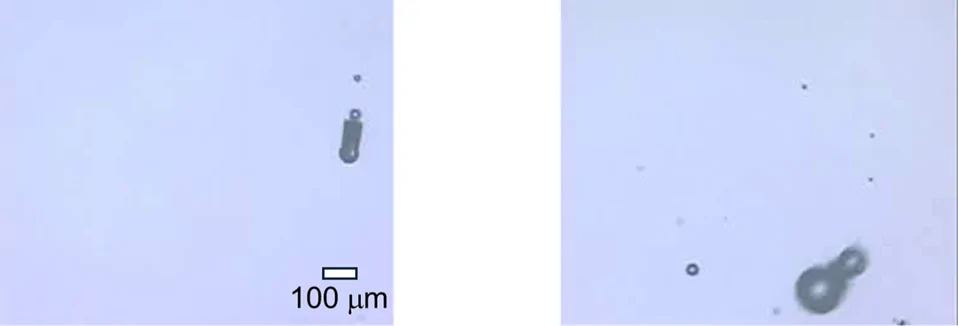
Baseline propulsion of the single-orifice micromotor. Left: 5 wt % H_2_O_2_ (73 μm s^–1^). Right: 15 wt % H_2_O_2_ (636 μm s^–1^). Videos: Movies S1 and S2.

**Table 2:** Representative Dimensions of the Single-Orifice Micromotor

dimension	design (μm)	measured (μm)^a^
total length	80	166
top width	35	90
base width	40	122
cavity height	—	∼1.33
stack thickness	—	∼2.71

a The measured values exceed the design values owing to lateral metal overgrowth during electrodeposition and slight expansion upon release from the substrate.

### 3.3 Baseline Propulsion: Single-Orifice Micromotor

The single-orifice micromotor, which served as the baseline (Figure 3; Movies S1 and S2), had a propulsion speed of 73 μm s^–1^ at 5 wt % H_2_O_2_, whereas that at 15 wt % was 636 μm s^–1^ (Table 3), an 8.7-fold enhancement. This dependence is consistent with conical-tube^5^ and roll-up^14^ micromotors, in which a higher fuel concentration increases the catalytic reaction rate and bubble nucleation frequency.^16,24^

**Table 3:** Summary of Propulsion Performance

configuration	condition	speed (μm s^–1^)	motion
single-orifice	5 wt % H_2_O_2_, uniform	73	directional
single-orifice	15 wt % H_2_O_2_, uniform	636	directional
two-orifice	15 wt % H_2_O_2_, uniform	∼450	curved
three-orifice^a^	10 wt % H_2_O_2_, uniform	not determined	straight
three-orifice^a^	H_2_O_2_ gradient (gel)	not determined	toward low-conc.

a For the three-orifice configuration, the analysis focused on the trajectory linearity and thrust-vector balance, which were quantified by tracking the micromotor centroid (Figure S8). Although the microscope was generally operated at 15 fps, the archived three-orifice clips did not retain the spatial calibration (e.g., an embedded scale bar) required for absolute speed determination, and the original calibration information is no longer accessible; the three-orifice speed is therefore listed as “not determined”, and only a scale-independent trajectory-linearity metric is reported.

### 3.4 Symmetry-dependent Motion: Two- vs Three-Orifice Micromotors

Prior studies on bubble-driven micromotors examined the propulsion behavior primarily as a function of the tube geometry (length, diameter, and semicone angle), catalytic surface area, and chemical environment.^14–17,24^ Template-electrodeposited conical micromotors^5,8^ and strain-driven roll-up tubular systems^10,12,13^ have revealed quantitative relationships between the geometric parameters and propulsion speed; however, all these architectures share a common feature: a single axially symmetric exhaust port. These previous investigations therefore focused on the extent to which the channel geometry modulates the propulsion speed and efficiency within the single-orifice regime. The question relating to the effect of the spatial arrangement of multiple exhaust ports on the motion state of the micromotor has therefore remained unanswered. None of the previous studies has compared different exhaust port configurations produced on the same fabrication platform with identical constituent metals and catalytic surface chemistry. This study addresses this limitation by isolating the effect of the number of exhaust ports and symmetry from the material and chemical variables by using a single lithographic platform to produce micromotors with one, two, and three orifices that differ only in terms of the geometry of their inner-layer photomasks.

Figure 4 shows the central experimental validation of the thrust symmetry concept by directly comparing the two-orifice and three-orifice motions of micromotors powered by fuel with a uniform concentration (i.e., the absence of a concentration gradient).

**Figure 4:**
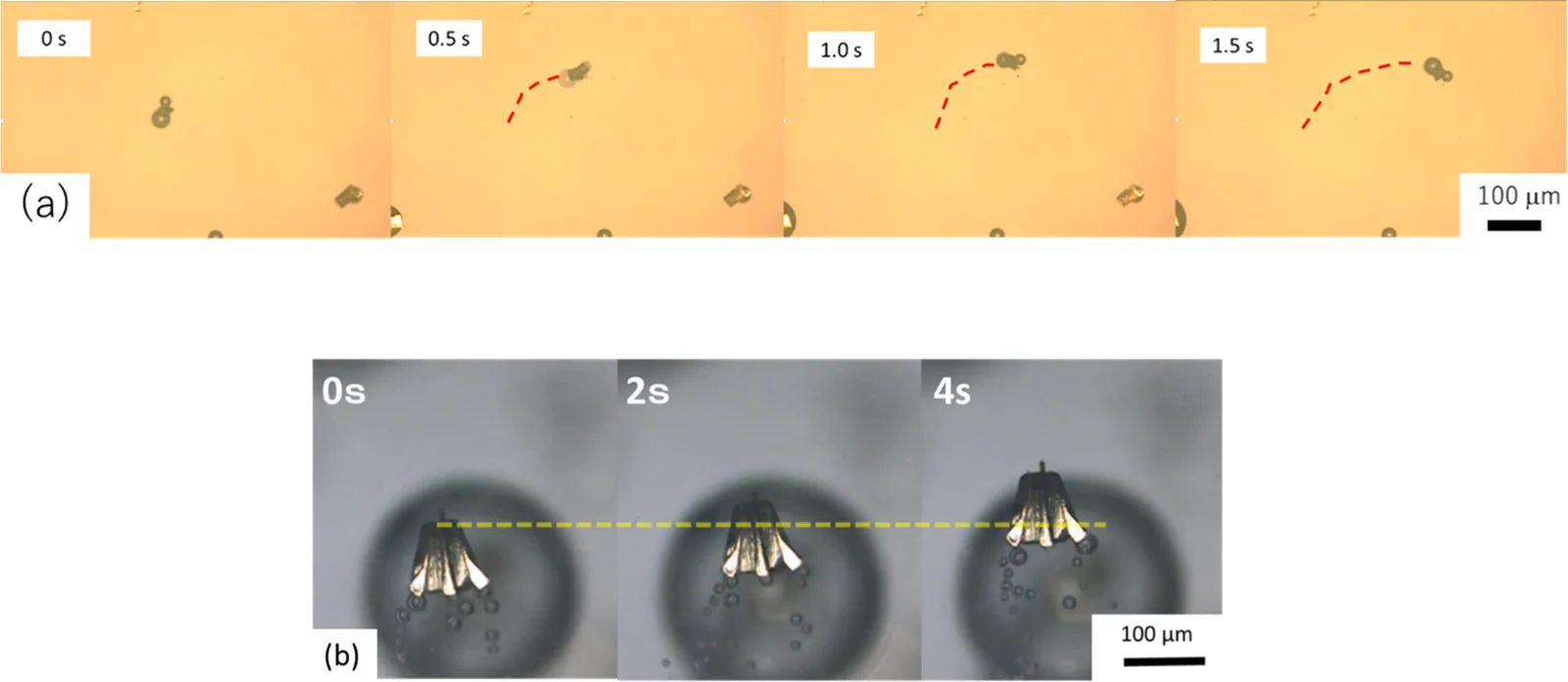
Symmetry-dependent motion: the central experimental validation. (a) Two-orifice micromotor in uniform 15 wt % H_2_O_2_: curved trajectory measured at 0.5 s intervals (∼450 μm s^–1^; Movie S4). (b) Three-orifice micromotor in uniform 10 wt % H_2_O_2_: straight-line trajectory measured at 2.0 s intervals (Movie S5). Trajectory linearity was quantified over the full observation window by tracking the micromotor centroid; the root-mean-square lateral deviation from the net-displacement axis is only about 7% of the net forward displacement, confirming rectilinear motion (see Figure S8 for the time-lapse panel and the centroid trajectory).

### 3.5 Two-Orifice System

In uniform 15 wt % H_2_O_2_, the two-orifice micromotor exhibited persistent curved trajectories at ∼450 μm s^–1^ (Figure 4a and Movie S4), as confirmed by trajectory analysis at 0.5 s intervals. This result reveals the intrinsic sensitivity of multiorifice thrust systems to geometric asymmetry; even a small deviation from perfect mirror symmetry between the two exhaust ports generates measurable and persistent net torque.^15,17^ The curved trajectory is the physically expected outcome of disrupted thrust-vector symmetry; any difference in the catalytic surface area, cross section of a port, or the length of the channel between the two ports produces thrust of unequal magnitude, and the resultant lateral force component drives continuous rotation. This establishes that multiorifice bubble micromotors operate in a regime in which small geometric deviations have macroscopically observable consequences for the motion state. More generally, rather than being set by bubble ejection alone, the resultant force and torque reflect a combined gas–liquid interfacial process: the port layout influences where bubbles grow, detach, and are ejected, and thereby shapes the local force generation at each port. In these terms, gas generation and bubble growth set the local availability of bubble thrust at each port, whereas bubble detachment and ejection set the direction and timing of momentum transfer, and interfacial capillary effects modulate the local release behavior; the observed curvature of the two-orifice motor and the straightening of the three-orifice motor are therefore interpreted as the macroscopic consequence of unequal versus balanced port-level bubble events rather than of a single isolated mechanism.

### 3.6 Three-Orifice System

The three-orifice design addresses this sensitivity by separating the thrust architecture into functionally distinct elements: a central port for forward propulsion and two lateral ports positioned symmetrically about the forward axis. Fabricated using μPG501 for the submicrometer overlay, the three-orifice micromotor exhibited straight-line motion in uniform 10 wt % H_2_O_2_ (Figure 4b and Movie S5), as confirmed by frame-by-frame tracking of the micromotor centroid over the full observation window (Figure S8). The trajectory is rectilinear: the root-mean-square lateral deviation from the net-displacement axis is only about 7% of the net forward displacement, in contrast to the persistent curvature of the two-orifice motor. Although the microscope was generally operated at 15 fps, the archived three-orifice clip did not retain the spatial calibration required to convert pixel displacements into absolute distances, so an absolute translational speed is not listed in Table 3; we therefore report the scale-independent trajectory-linearity metric instead. The observed straight motion is consistent with the balanced lateral thrust for mirror-symmetric port placement.^17^

The contrast between persistent curved motion (two-orifice, broken symmetry) and reproducible straight motion (three-orifice, restored symmetry) driven by fuel with a uniform concentration constitutes the core finding. Within the present platform, while maintaining the same material composition and catalytic interface, the exhaust-port layout acts as an important geometric control parameter for whether the motion remains curved or becomes rectilinear.^25^

The difference in motion between the two- and three-orifice micromotors is unlikely to be governed by exhaust-port symmetry alone. The two configurations differ not only in port arrangement but also in mass distribution, projected area, rotational (mass) moment of inertia, and hydrodynamic resistance to rotation. Because the three-orifice device carries its lateral ports and additional material farther from the central axis, its larger rotational inertia and rotational drag can reduce the angular response to a given torque, which would also favor straighter trajectories. Consistent with this, a recent analysis of bubble-driven Janus micromotors reported that smaller motors are more likely to follow straight paths, with the nucleated bubble contributing both a propulsive force and a rotational torque.^26^ Our results should therefore be read not as evidence that exhaust-port symmetry is the sole determinant of the motion state, but as showing that, under fixed material and catalytic conditions, the exhaust-port layout strongly affects the observed propulsion state. At the same time, the directional deflection of the three-orifice motor under a H_2_O_2_ concentration gradient (Section 3.5) indicates that the three-orifice geometry is not merely a more rotation-resistant rigid body: differential bubble generation at the lateral ports can still redirect the motion.

### 3.7 Motion Response in a Non-Uniform Fuel Environment

The three-orifice geometry was designed not only to recover straight-line motion under uniform conditions but also to geometrically transduce spatial variations in the fuel concentration into directional motion. In this architecture, the central port provides a stabilizing forward thrust that maintains the overall propulsion, while the two lateral ports act as differential response elements whose local bubble-generation rates vary with the local H_2_O_2_ concentration. This separation of functions is a direct consequence of the port layout. Under a nonuniform fuel environment, the local rate of bubble generation and growth is expected to differ across the lateral ports, biasing the subsequent detachment and ejection events and thus the resulting thrust composition, which is consistent with the directional, gradient-biased response observed here.

To test this, a H_2_O_2_-loaded agar gel was placed 2 cm from the observation field. In this nonuniform environment, the three-orifice micromotor was propelled toward the low-concentration region away from the gel source (Figure 5a and Movie S6). This behavior is consistent with the differential bubble generation at the two lateral ports, which produces a net lateral thrust and thereby deflects the trajectory. The negative control, prepared with only Au particles (same geometry, but without Pt) did not demonstrate directed motion (Figure 5b), confirming that the response requires active catalytic propulsion.

**Figure 5:**
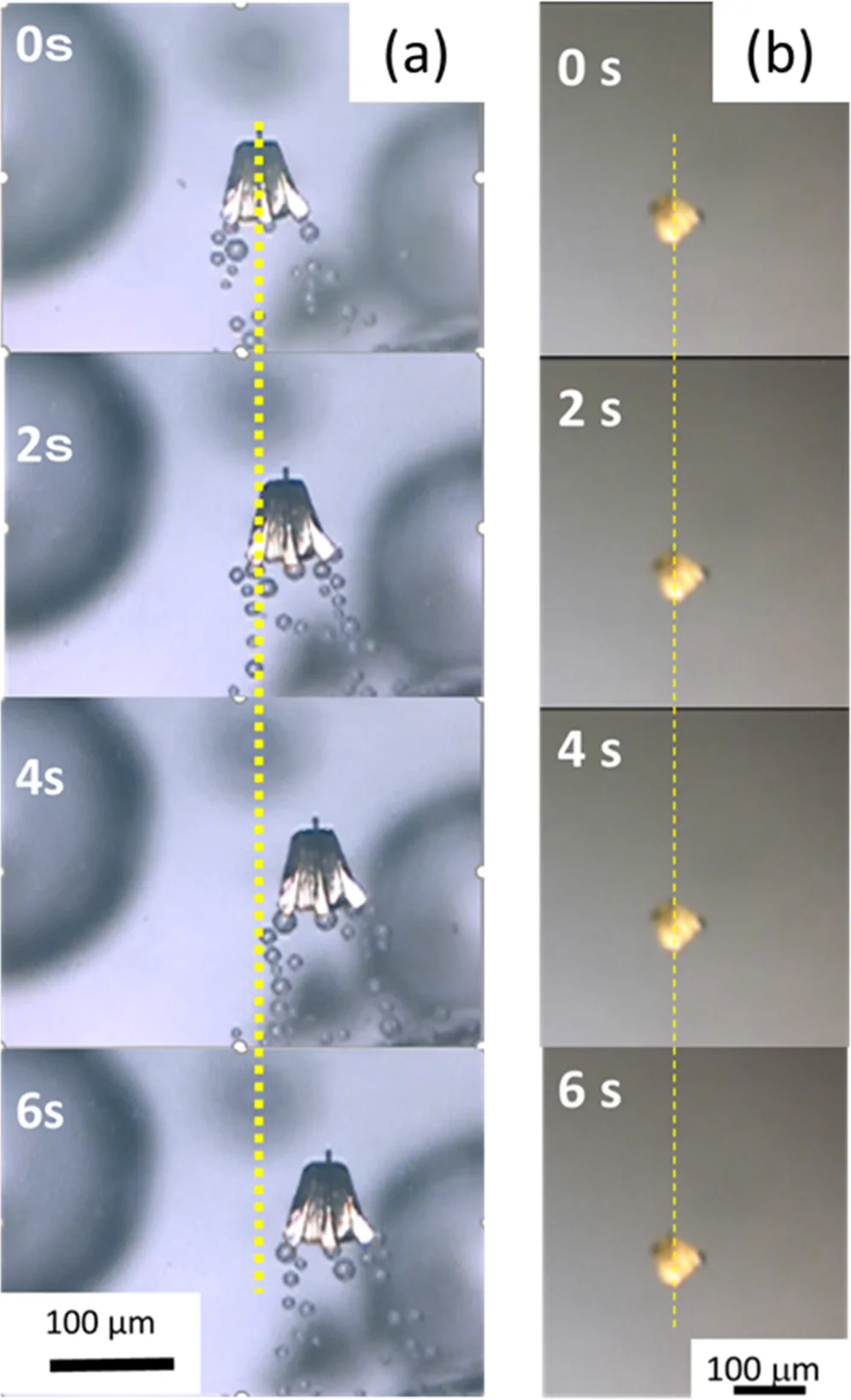
Motion response in a nonuniform fuel environment. (a) Three-orifice micromotor trajectory (2.0 s intervals) with H_2_O_2_ gel placed to one side; motion directed toward the low-concentration side (Movie S6). (b) Au-only negative control under identical gradient conditions—no directed motion observed. A clearer bubble-free Au-only control frame was not available from the present archived image set; therefore, the original control image was retained with clarified labeling.

The observed directional response is interpreted as a geometry-mediated transduction of environmental heterogeneity; a spatial fuel gradient produces differential bubble generation rates at the lateral ports, which creates a thrust imbalance that deflects the trajectory, whereas the central port maintains forward propulsion. This behavior is reminiscent of, but mechanistically distinct from, biological chemotaxis.^27–29^ Critically, the system possesses no sensing mechanism, no signal transduction pathway, and no adaptive response; directional motion arises entirely from passive differential catalysis acting on a geometrically defined multiport structure. Therefore, the observed behavior constitutes a geometry-mediated transduction of environmental heterogeneity rather than an active tactic response. Accordingly, we describe this as a motion response in a nonuniform fuel environment. This minimal, geometry-mediated bias of the trajectory in a fuel gradient, which we refer to as a gradient-biased motion response, can be regarded as a basic functional response to an environmental gradient; it is distinct from biological chemotaxis precisely because it involves no sensing, signal transduction, or adaptation. The primary aim of the present study is to establish the design principle by which the multiorifice exhaust-port layout sets the propulsion state, rather than to demonstrate a complete application. Nevertheless, combining the programmable port layout with this gradient-biased response suggests a route toward simple functional tasks such as targeted delivery, micromanipulation, environmental remediation, and microsensing, building on capabilities reported for other bubble-driven microrobots.^20–23^

### 3.8 Design Principle: Geometric Control of the Propulsion State

The results established a design principle we termed thrust symmetry engineering. Within the present platform and under fixed material and catalytic conditions, the geometric layout of the exhaust ports acts as an important geometric control parameter for the observed propulsion state. Three regimes were identified: (i) single orifice: directional thrust along the channel axis, with the speed scaling with the fuel concentration; (ii) multiorifice with broken symmetry: the net torque produces curved trajectories whose curvature reflects the degree of geometric asymmetry; and (iii) multiorifice with mirror symmetry: the lateral thrust components cancel each other to restore straight-line motion, whereas an externally imposed concentration gradient can introduce a controlled left–right imbalance through differential bubble generation at the lateral ports.

This geometric approach complements existing strategies for micromotor motion control that have relied primarily on external fields^11,18^ or fuel-chemistry modification.^24^ The lithographic platform ensures that this geometric control is practically applicable: the inner-layer photomask independently specifies the exhaust port layout, while all other process parameters, including the inner surface of the Pt catalyst and the Au structural shell, remain fixed.^30^

A key contribution of the present work is the experimental isolation of the geometric effects of the material and the interfacial variables. By holding the constituent metals (Au/Pt), catalytic inner-surface chemistry, and fuel system constant while varying only the exhaust port layout, this study demonstrates that, within the present platform and under fixed catalytic and material conditions, the geometry of the exhaust port can switch the propulsion mode among directional, curved, and gradient-biased motion responses. This isolation could not be realized in previous single-orifice tubular or conical micromotor studies, in which the geometry and was typically coupled to the type of material. The results indicate that the catalytic inner surface and exhaust port geometry together constitute an interface-programmed propulsion architecture in which the motion state is encoded in the lithographically defined port layout rather than in the material composition.^13–15^

## 4 Conclusions

We demonstrated that, within the present platform and under fixed material and catalytic conditions, the propulsion behavior of bubble-driven micromotors can be programmed by designing the geometry of the catalytic exhaust port layout, a principle we termed thrust symmetry engineering. The single-orifice baseline exhibits concentration-dependent propulsion (73 μm s^–1^ at 5 wt % H_2_O_2_; 636 μm s^–1^ at 15 wt %). The two-orifice system reveals that even small geometric asymmetries produce a persistent trajectory curvature, demonstrating the intrinsic sensitivity of the multiport thrust to symmetry breaking. The three-orifice system restores the straight-line motion through mirror-symmetric port placement and additionally exhibits directional motion in a nonuniform fuel environment via differential bubble generation at functionally distinct lateral ports. For the same Au/Pt structural shell, catalytic inner-surface chemistry, and fuel system, variation of the port layout in this study enabled the geometric effects to be experimentally isolated from the material and interfacial variables. This allowed the exhaust port geometry to be identified as an important geometric control parameter for tuning the propulsion mode within the investigated system. Thus, the lithographic platform provides an interface-programmable architecture for active catalytic micromotor design, in which the motion state is encoded in the port layout rather than in the composition of the material. This approach offers a scalable route for programmable catalytic transport with potential applications in environmental remediation^31,32^ and targeted delivery.^33^

## Supplementary Material















## Notes

Single-orifice micromotor in 5 wt % H_2_O_2_ (73 μm s^–1^) (WMV)
Single-orifice micromotor in 15 wt % H_2_O_2_ (636 μm s^–1^) (WMV)
*D*_in_ ≈ *D*_out_ variant in 15 wt % H_2_O_2_ (no propulsion) (WMV)
Two-orifice micromotor in uniform 15 wt % H_2_O_2_ (curved trajectory, ∼450 μm s^–1^) (MP4)
Three-orifice micromotor in uniform 10 wt % H_2_O_2_ (straight-line motion) (MP4)
Three-orifice micromotor under an H_2_O_2_ concentration gradient established by an agar gel source placed to the left of the observation field. The micromotor moves in the direction opposite to the gel source (i.e., toward the low-concentration side), consistent with differential bubble generation at the lateral ports. The second half of the video shows the Au-only negative control (same three-orifice geometry, no Pt catalyst) under identical gradient conditions, exhibiting no directed motion (MP4)
Photomask designs (Figure S1), theoretical speed model (Figure S2), electrochemical cell (Figure S3), deposition conditions (Figure S4), stage-by-stage images (Figure S5), cross-sectional SEM/profilometry (Figure S6), *D*_in_ ≈ *D*_out_ variant (Figure S7), fabrication reproducibility and statistics (Table S1), extended trajectory analysis of the three-orifice micromotor (Figure S8) (PDF)

## §

Subaru Corporation, 1-1, Subaru-cho, Ota-shi, Gunma 373-8555, Japan

## ∥

DENSO Corporation, Electronics Engineering Division 1, 5-1403 Kamiyashiro, Meito-ku, Nagoya, Aichi 465-0025, Japan.

## ⊥

Department of Engineering, Graduate School of Engineering, Nagoya Institute of Technology, Gokiso-cho, Showa-ku, Nagoya 466-8555, Japan.

## Abbreviations

Ag: silver
Au: gold
CE: counter electrode
Cu: copper
FE-SEM: field-emission scanning electron microscopy
HMDS: hexamethyldisilazane
H_2_O_2_: hydrogen peroxide
O_2_: oxygen
PPE: personal protective equipment
PR: photoresist
Pt: platinum
RE: reference electrode
RT: room temperature
WE: working electrode

## References

[1] Wang H., Pumera M. (2015). Fabrication of micro/nanoscale
motors. Chem. Rev..

[2] Gao W., Wang J. (2014). The environmental impact of micro/nanomachines: A review. ACS Nano.

[3] Sánchez S., Soler L., Katuri J. (2015). Chemically
powered micro- and nanomotors. Angew. Chem.,
Int. Ed..

[4] Li J., Esteban-Fernández
de Ávila B., Gao W., Zhang L., Wang J. (2017). Micro/nanorobots
for biomedicine: Delivery, surgery, sensing, and
detoxification. Sci. Robot..

[5] Gao W., Sattayasamitsathit S., Orozco J., Wang J. (2011). Highly efficient catalytic
microengines: Template electrosynthesis of polyaniline/platinum microtubes. J. Am. Chem. Soc..

[6] Gao W., Wang J. (2014). Synthetic micro/nanomotors
in drug delivery. Nanoscale.

[7] Wrede P., Medina-Sánchez M., Fomin V. M., Schmidt O. G. (2021). Switching
propulsion mechanisms of tubular catalytic micromotors. Small.

[8] Gao W., Sattayasamitsathit S., Uygun A., Pei A., Ponedal A., Wang J. (2012). Polymer-based
tubular microbots: Role of composition and preparation. Nanoscale.

[9] Wang J. (2013). Template electrodeposition
of catalytic nanomotors. Faraday Discuss..

[10] Solovev A. A., Mei Y., Bermúdez Ureña E., Huang G., Schmidt O. G. (2009). Catalytic
microtubular jet engines self-propelled by accumulated gas bubbles. Small.

[11] Solovev A. A., Sanchez S., Pumera M., Mei Y. F., Schmidt O. G. (2010). Magnetic
control of tubular catalytic microbots for the transport, assembly,
and delivery of micro-objects. Adv. Funct. Mater..

[12] Mei Y., Huang G., Solovev A. A., Ureña E. B., Mönch I., Ding F., Reindl T., Fu R. K. Y., Chu P. K., Schmidt O. G. (2008). Versatile approach for integrative
and functionalized tubes by strain engineering of nanomembranes on
polymers. Adv. Mater..

[13] Mei Y., Solovev A. A., Sanchez S., Schmidt O. G. (2011). Rolled-up nanotech
on polymers: From basic perception to self-propelled catalytic microengines. Chem. Soc. Rev..

[14] Li J., Huang G., Ye M., Li M., Liu R., Mei Y. (2011). Dynamics of catalytic tubular microjet
engines: Dependence on geometry
and chemical environment. Nanoscale.

[15] Fomin V. M., Hippler M., Magdanz V., Soler L., Sanchez S., Schmidt O. G. (2014). Propulsion mechanism of catalytic microjet engines. IEEE Trans. Robot..

[16] Zhao G., Nguyen N.-T., Pumera M. (2013). Reynolds numbers
influence the directionality
of self-propelled microjet engines in the 10(−4) regime. Nanoscale.

[17] Li L., Wang J., Li T., Song W., Zhang G. (2014). Hydrodynamics
and propulsion mechanism of self-propelled catalytic micromotors:
Model and experiment. Soft Matter.

[18] Li J., Gao W., Dong R., Pei A., Sattayasamitsathit S., Wang J. (2014). Nanomotor lithography. Nat. Commun..

[19] Yang Q., Xu L., Zhong W., Yan Q., Gao Y., Hong W., She Y., Yang G. (2020). Recent advances in motion control of micro/nanomotors. Adv. Intell. Syst..

[20] Xu Z., Sun H., Chen Y., Yu H. H., Deng C.-X., Xu Q. (2024). Bubble-inspired
multifunctional magnetic microrobots for integrated multidimensional
targeted biosensing. Nano Lett..

[21] Wang L., Sheng M., Chen L., Yang F., Li C., Li H., Nie P., Lv X., Guo Z., Cao J., Wang X., Li L., Hu A. L., Guan D., Du J., Cui H., Zheng X. (2024). Sub-nanogram resolution measurement
of inertial mass and density using magnetic-field-guided bubble microthruster. Adv. Sci..

[22] Wang L., Chen L., Zheng X., Yu Z., Lv W., Sheng M., Wang L., Nie P., Li H., Guan D., Cui H. (2022). Multimodal bubble microrobot near
an air–water interface. Small.

[23] Dai L., Lin D., Wang X., Jiao N., Liu L. (2020). Integrated
assembly
and flexible movement of microparts using multifunctional bubble microrobots. ACS Appl. Mater. Interfaces.

[24] Zhao G., Sanchez S., Schmidt O. G., Pumera M. (2013). Poisoning
of bubble
propelled catalytic micromotors: The chemical environment matters. Nanoscale.

[25] Ren L., Wang W., Mallouk T. E. (2018). Two forces
are better than one: Combining
chemical and acoustic propulsion for enhanced micromotor functionality. Acc. Chem. Res..

[26] Wang L., Li H., Liu Z., Li C., Zhang J., Chen L., Wang X., Guan D., Zheng X., Cui H. (2025). Is the circular
motion of bubble-driven spherical Janus micromotors deterministic
or random?. Phys. Fluids.

[27] Gao C., Feng Y., Wilson D. A., Tu Y., Peng F. (2022). Micro-nano
motors with taxis behavior: Principles, designs, and biomedical applications. Small.

[28] Hong Y., Blackman N. M. K., Kopp N. D., Sen A., Velegol D. (2007). Chemotaxis
of nonbiological colloidal rods. Phys. Rev.
Lett..

[29] Baraban L., Harazim S. M., Sanchez S., Schmidt O. G. (2013). Chemotactic behavior
of catalytic motors in microfluidic channels. Angew. Chem., Int. Ed..

[30] Safdar M., Khan S. U., Jänis J. (2018). Progress toward
catalytic micro-
and nanomotors for biomedical and environmental applications. Adv. Mater..

[31] Soler L., Magdanz V., Fomin V. M., Sanchez S., Schmidt O. G. (2013). Self-propelled
micromotors for cleaning polluted water. ACS
Nano.

[32] Jurado-Sánchez B., Wang J. (2018). Micromotors
for environmental applications: A review. Environ.
Sci.: Nano.

[33] de
Ávila B. E.-F., Angsantikul P., Li J., Angel Lopez-Ramirez M., Ramírez-Herrera D. E., Thamphiwatana S., Chen C., Delezuk J., Samakapiruk R., Ramez V., Obonyo M., Zhang L., Wang J. (2017). Micromotor-enabled
active drug delivery for in vivo treatment of stomach infection. Nat. Commun..

