# Supplementary material for: Thrust Symmetry Engineering in Lithographically Defined Bubble-Propelled Micromotors via Multi-Orifice Exhaust-Port Design
Source: ACS Omega. 2026 Aug 1;11(32):48463–71. doi: 10.1021/acsomega.6c05214 (PMC13491906; doi:10.1021/acsomega.6c05214)
Supplement: Supplementary file 1 [file ao6c05214_si_007.pdf]

Supporting Information

for

Thrust Symmetry Engineering in Lithographically Defined Bubble-Propelled Micromotors via Multi-Orifice Exhaust-Port Design

Isao Shitanda[a,b*,‡], *Takuma Suzuki*[a,‡,§], *Kentaro Zama*[a,‡,¶], *Yoshinao Hoshi*[a,†], *Masanori Hayase*[a,b], *Masayuki Itagaki*[a,b]

[a]Department of Pure and Applied Chemistry, Faculty of Science and Technology, Tokyo University of Science, 2641, Yamazaki, Noda, Chiba 278-8510, Japan

[b]Research Institute for Science and Technology, Tokyo University of Science, 2641 Yamazaki, Noda, Chiba 278-8510, Japan

§ Present address: SUBARU CORPORATION, 1-1, Subaru-cho, Ota-shi, Gunma 373-8555, Japan

¶ Present address: DENSO CORPORATION, Electronics Engineering Division 1, 5-1403 Kamiyashiro, Meito-ku, Nagoya, Aichi 465-0025, Japan

† Present address: Department of Engineering, Graduate School of Engineering, Nagoya Institute of Technology, Gokiso-cho, Showa-ku, Nagoya 466-8555, Japan

*Corresponding author. Tel.: +81 4 7124 1401; Fax: +81 4 7123 9890.

‡ Equal contribution

E-mail address: shitanda@rs.noda.tus.ac.jp (I. Shitanda).

Contents

Figure S1. Photomask designs for single configurations.
Figure S2. Theoretical propulsion speed model and geometry design rationale.
Figure S3. Electrochemical cell configuration and experimental apparatus.
Figure S4. Detailed deposition conditions for Au, Pt electrodeposition and Pt sputtering.
Figure S5. Optical microscopy images at each fabrication stage.
Figure S6. Cross-sectional SEM and laser profilometry of the single-orifice baseline.
Figure S7. Alternative geometry variant ($D_{in} \approx D_{out}$) propulsion test.
Figure S8. Full-clip centroid tracking and scale-independent linearity analysis of the three-orifice micromotor.

Table S1. Available geometric and motion-analysis information from archived data.

Note S1. Basis for speed quantification in the retained video clips.

Movies S1–S6. Propulsion videos for single-, two-, and three-orifice micromotors.

Figure S1. Photomask Designs

The photomask patterns define the outer-layer footprint and inner-layer void geometry for each micromotor configuration. For the single-orifice system, the outer layer is trapezoidal (35/40/80 µm) and the inner layer defines a single void (D_in: 7 µm, D_out: 14 µm, height: 112 µm). For the two-orifice system, a modified V-shaped inner-layer pattern creates two diverging channels. For the three-orifice system, a three-pronged inner-layer pattern defines one central and two lateral channels.

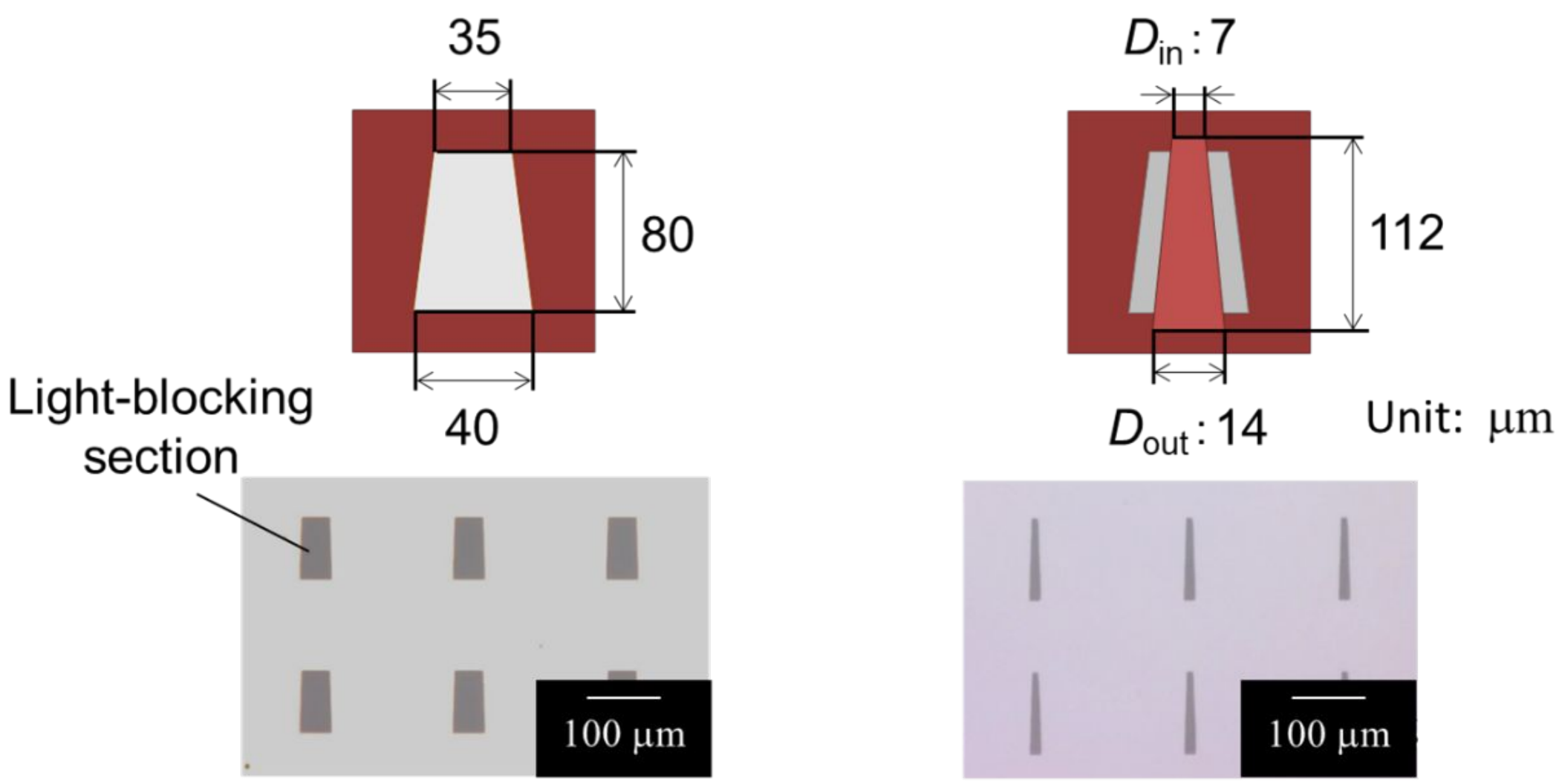

Figure S1. Photomask design drawings and optical images for the single-orifice configuration. Left: outer-layer photomask (35/40/80 µm). Right: inner-layer photomask (D_in: 7, D_out: 14 µm, height: 112 µm). Scale bars: 100 µm. Two- and three-orifice photomask designs follow the same fabrication logic with modified inner-layer geometries.

Figure S2. Theoretical Speed Model

The micromotor geometry was designed using the theoretical model by Zhao et al. (Nanoscale, 2013, 5, 7277). With L = 80 µm, parametric calculation of D_{in} and D_{out} indicated maximum speed when $D_{in} \approx D_{out}$, but practical propulsion requires $D_{in} \neq D_{out}$ to maintain directional asymmetry. The single-orifice design adopted $D_{in} \approx 7$ µm, $D_{out} \approx 14$ µm.

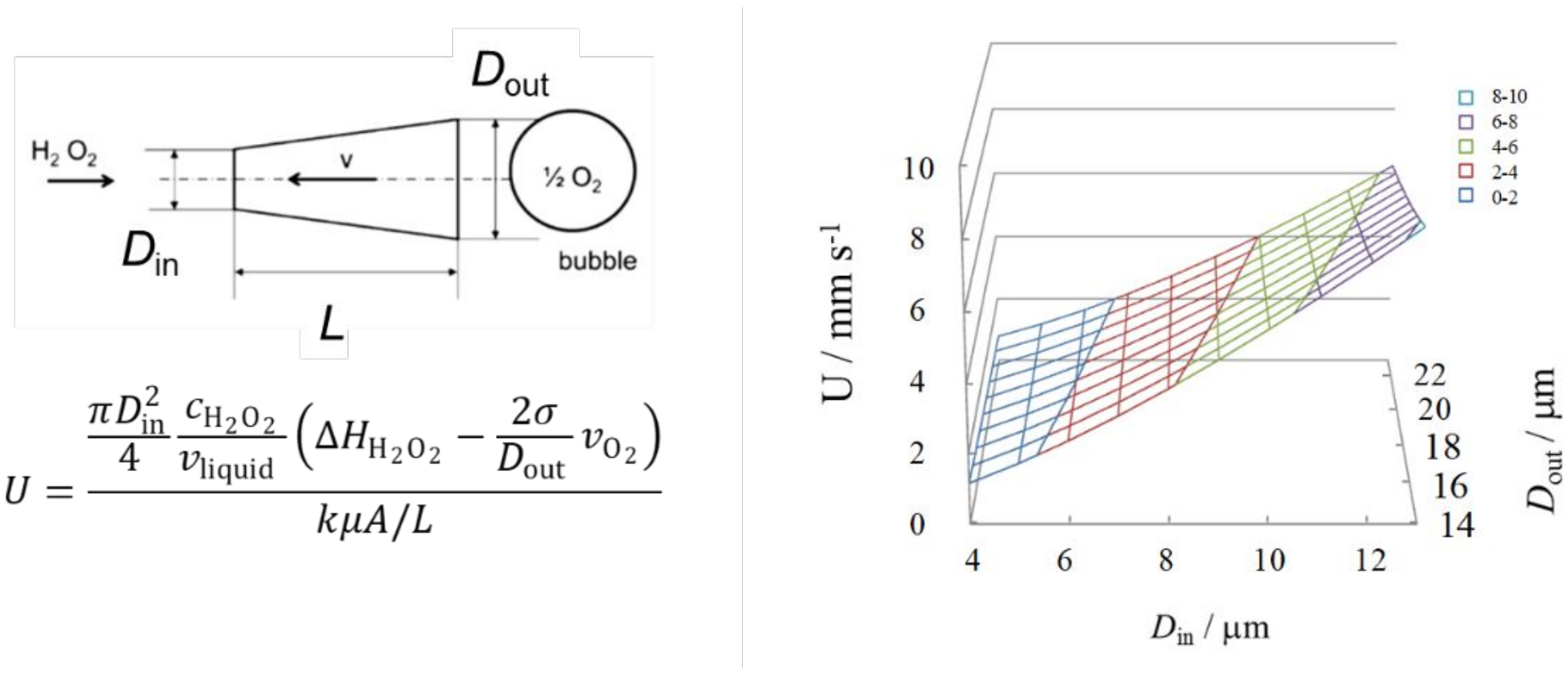

Figure S2. Propulsion speed model. Left: conical-tube model parameters. Right: calculated speed U vs. D_{in} and *D*out (L = 80 µm). v_{liquid}: specific volume of the liquid, v_{O_2}: specific volume of oxygen, σ: surface tension, k: correction factor, m: viscosity of the liquid, A: surface area of the micromotor

Figure S3. Experimental Apparatus

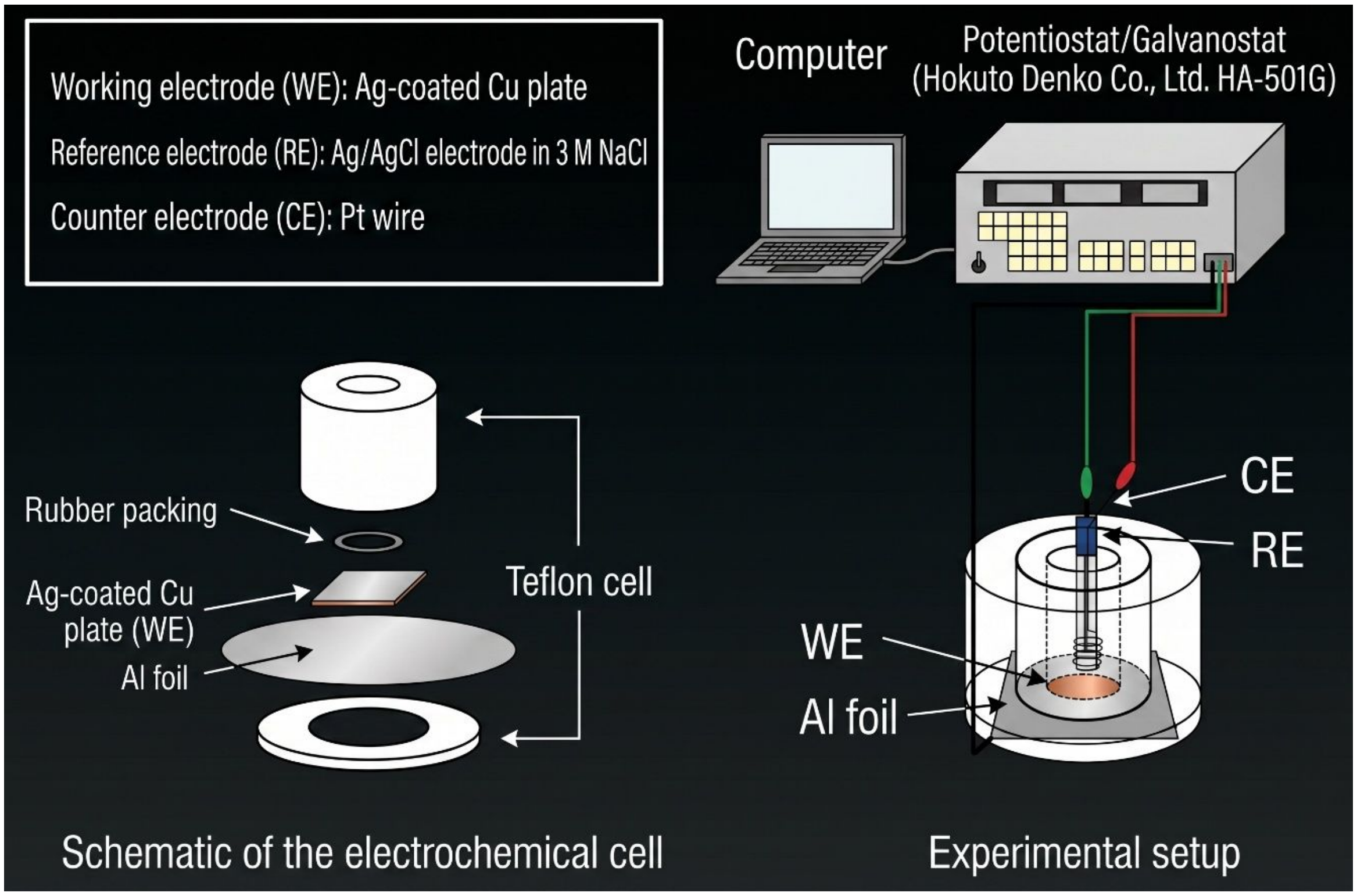

Figure S3. Electrochemical cell and experimental setup. Left: Teflon cell schematic (WE: Ag-coated Cu, RE: Ag/AgCl, CE: Pt wire). Right: complete apparatus with potentiostat/galvanostat (HA-501G, Hokuto Denko).

Figure S4. Deposition Conditions

Au Plating Solution (Product Data)

Manufactured by Nippon High Purity Chemicals Co., Ltd.
Temper Resist K-91H
pH: 7.3–7.8
Bath temperature: 50 ° C
Current density: -1.9×10^{-3} A/cm^2
Electroplating time: 72 sec

Pt Plating Solution (Product Data)

Manufactured by Mattex Japan Co., Ltd.
Platinassid PT-10
pH: 1.0 or less
Bath temperature: Roomtemperature
Current Density: -5.4×10^{-3} A/cm
Electroplating time: 90 sec

Pt Sputtering

Manufactured by San-Yu Electronics Co., Ltd.
QUICK COATER SC-701HMC2
Inlet gas: Ar, System pressure: 2.0 Pa, Discharge current: 20 mA,
Discharge voltage: 0.5 kV, Deposition time: 4 min 30 sec,
Film thickness: 100 nm

Figure S4. Deposition parameters. Au plating: K-91H, pH 7.3–7.8, 50 °C, -5.4×10^{-3} A cm^{-2}, 90 s. Pt plating: PT-10, pH < 1.0, RT, -1.9×10^{-3} A cm^{-2}, 72 s. Pt sputtering: SC-701HMC2, Ar, 2.0 Pa, 20 mA, 0.5 kV, 4 min 30 s, ~100 nm.

Figure S5. Stage-by-Stage Fabrication Images

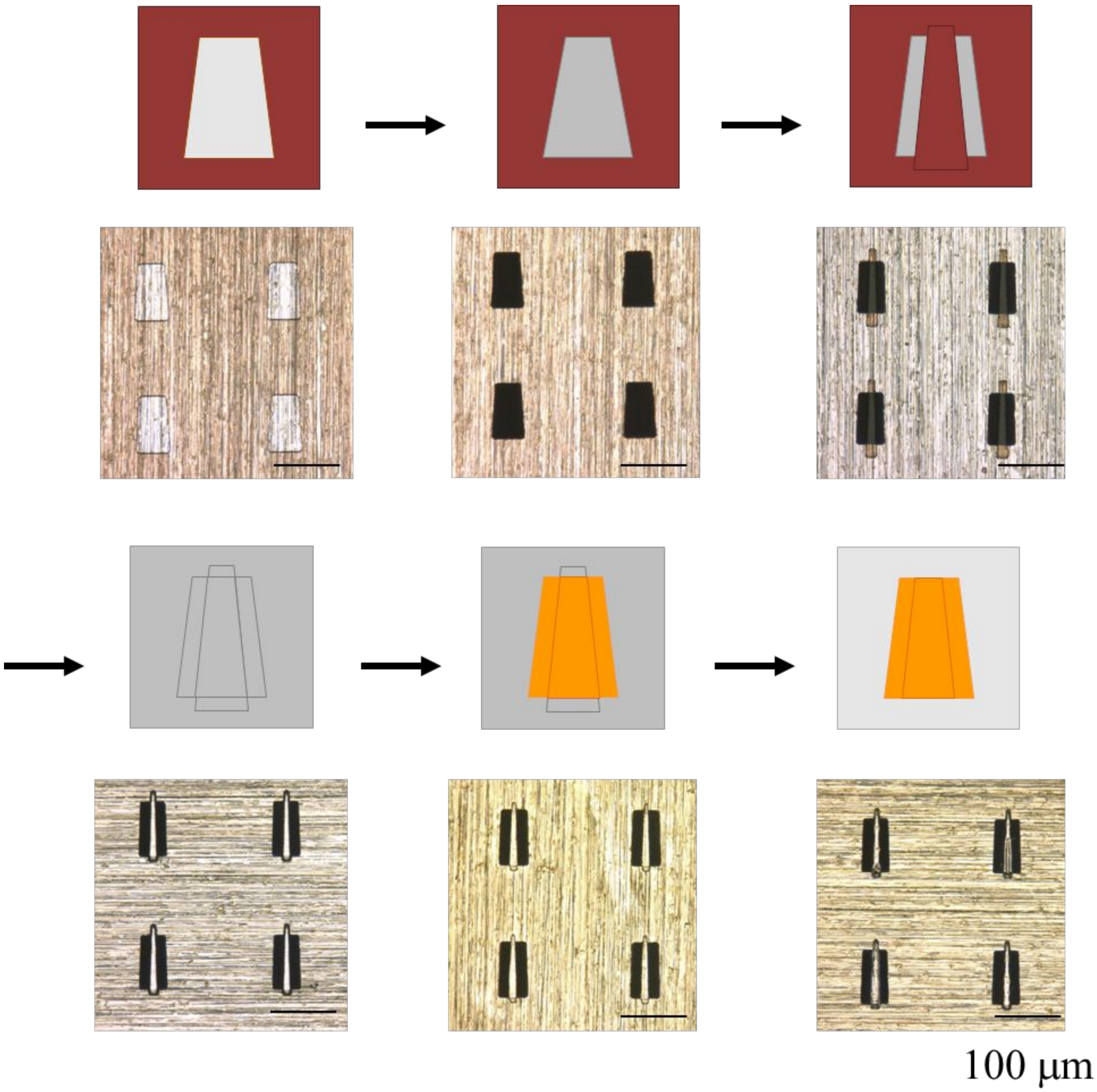

Figure S5. Optical images at each fabrication stage with corresponding process schematics. Scale bars: 100 µm.

Figure S6. Cross-Sectional Characterization

The single-orifice baseline micromotor was cross-sectioned and examined by FE-SEM and laser profilometry. The hollow internal cavity has a maximum height of ~1.33 µm within a total stack thickness of ~2.71 µm. The combined Au/Pt layer thickness is ~0.95 µm.

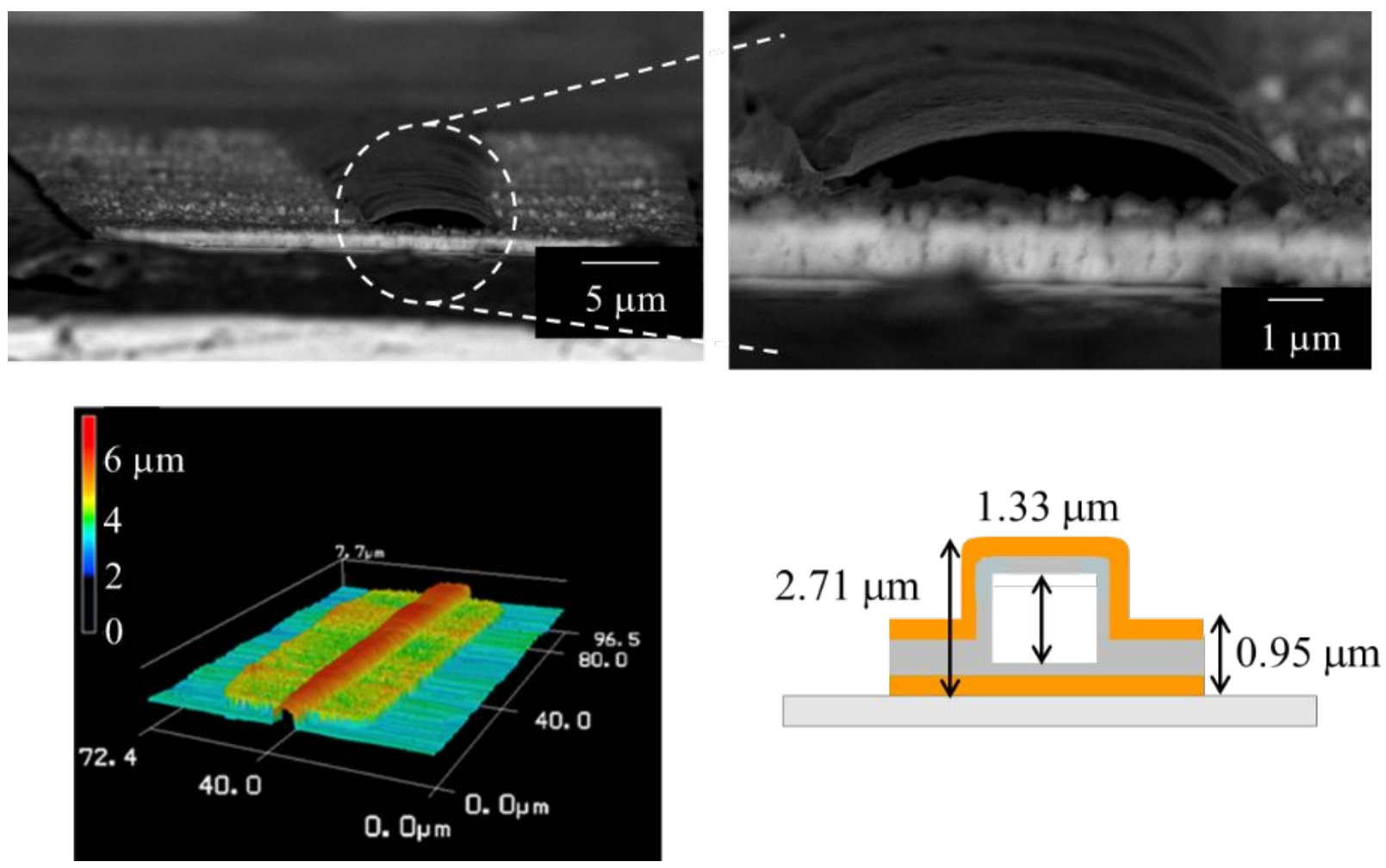

Figure S6. Cross-sectional characterization of the single-orifice stacked micromotor. Top: FE-SEM images (scale bars: 5 µm, 1 µm). Bottom left: laser microscopy 3D profile. Bottom right: thickness schematic (cavity: 1.33 µm, total: 2.71 µm, Au/Pt: 0.95 µm).

Figure S7. Alternative Geometry Variant ($D_{in} \approx D_{out}$)

An alternative single-orifice geometry with $D_{in} \approx D_{out}$ (12/14 µm) was fabricated and tested in 15 wt% H_2O_2. Despite successful fabrication and release, no forward propulsion was observed, consistent with the theoretical prediction that near-equal inlet and outlet diameters eliminate the net directional asymmetry required for bubble ejection. This result validates the importance of maintaining $D_{in} \neq D_{out}$.

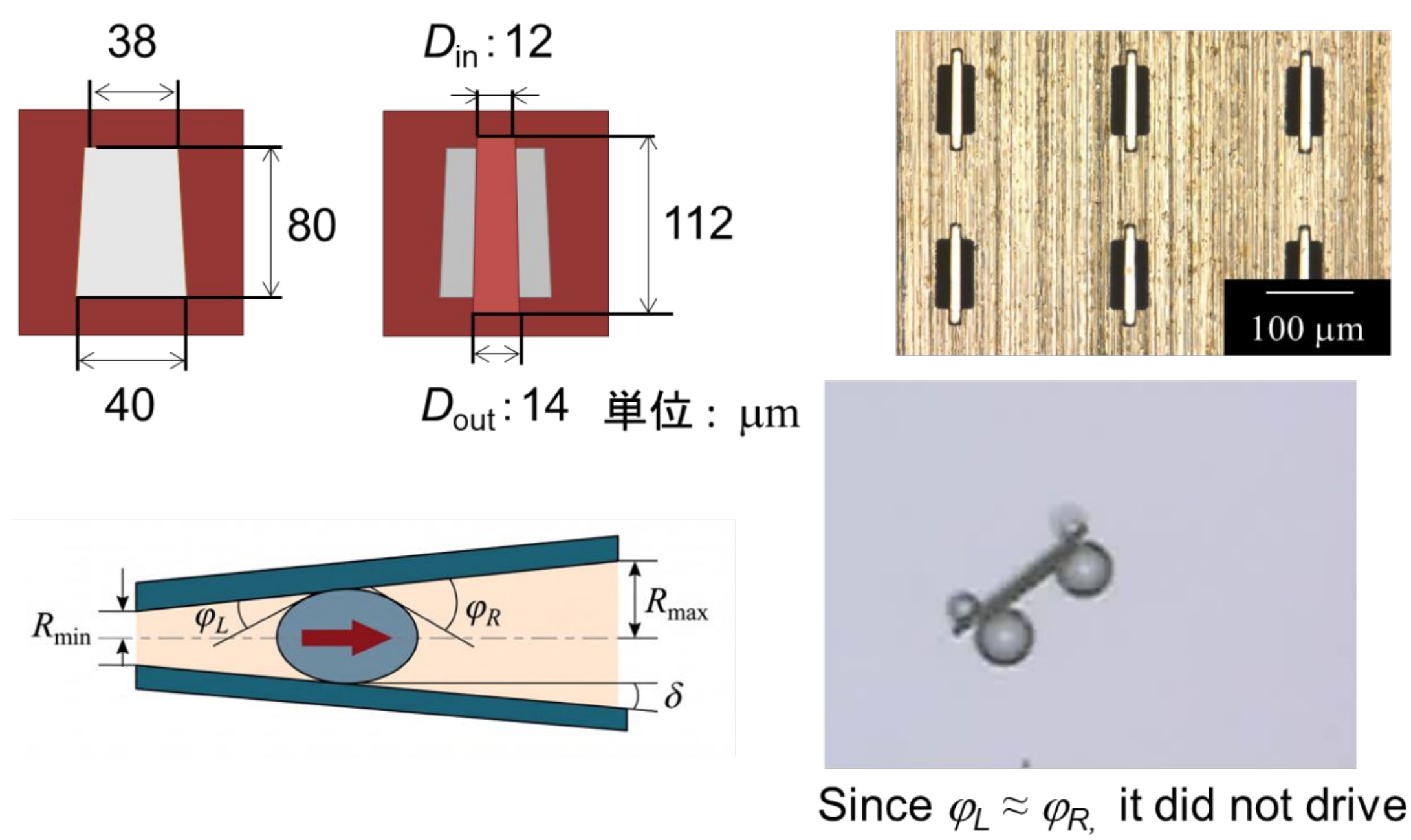

Figure S7. D_in ≈ D_out variant. Photomask: 38/40/80 µm outer; 12/14 µm inner. Fabricated micromotors (scale bar: 100 µm) showed bubble generation but no sustained forward propulsion ($L \approx R$ condition).

Figure S8. Extended Trajectory Analysis of the Three-Orifice Micromotor

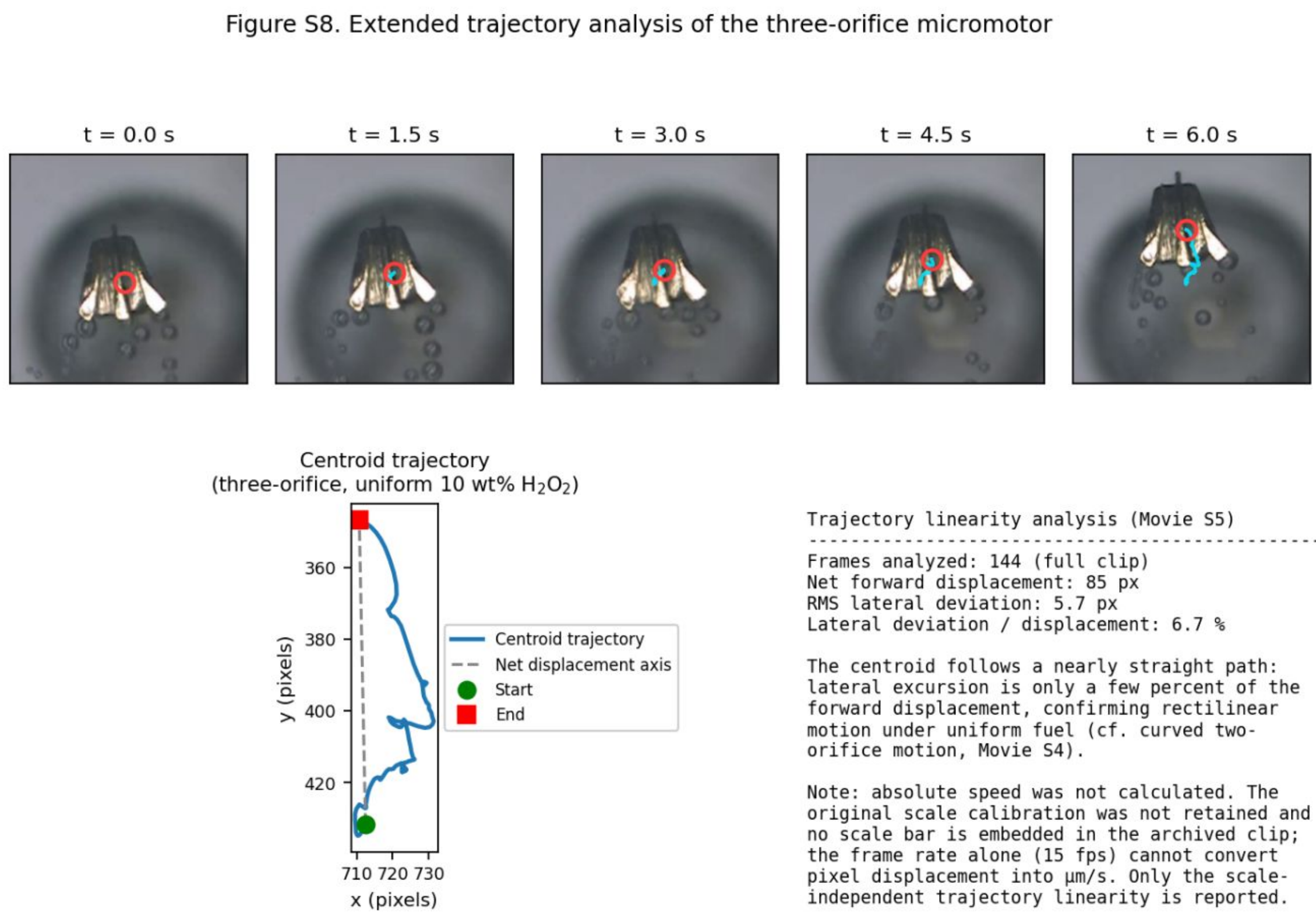

Figure S8. Extended trajectory analysis of the three-orifice micromotor in uniform 10 wt% H_2O_2 (Movie S5). Top: time-lapse snapshots at five time points spanning the clip, with the tracked micromotor centroid (red marker) and its trajectory (cyan) overlaid. Bottom left: centroid trajectory over the full window. Bottom right: quantitative summary. The root-mean-square lateral deviation from the net-displacement axis is only about 7% of the net forward displacement, confirming rectilinear motion, in contrast to the persistent curvature of the two-orifice motor (Movie S4). The absolute translational speed is not reported because the archived three-orifice clip does not retain the spatial (length) calibration required to convert displacements into absolute units, and no scale bar is embedded; although the microscope was generally operated at 15 fps, the frame rate alone is insufficient without the spatial calibration. The linearity metric reported here is scale-independent.

Table S1. Available Geometric and Motion-Analysis Information from Archived Data

This table summarizes the experimentally traceable geometric and motion-analysis information available from the archived images and videos for each configuration. The single-orifice dimensions are the measured values reported in the main text (Table 2). Complete batch-level fabrication counts and per-dimension standard deviations were not retained in the archived data set; to avoid reporting unsupported values, such counts are not tabulated, and only traceable information is listed. For each configuration, the archived dataset retained one analyzable representative device, which was used for the motion analysis reported in the manuscript; this is indicated as n in the table. Batch-level fabrication counts were not part of the retained records and are therefore not tabulated; no unsupported counts are reported.

**Configuration**	**Analyzable devices, n**	**Available archived record**	**Representative geometric information**	**Motion-analysis record**	**Notes**
Single-orifice	1	Optical micrographs (Figures 3 and S5); Movies S1 and S2	Length 166 µm, top width 90 µm, base width 122 µm (Table 2); inner void D_{in} 7 µm, D_{out} 14 µm, height 112 µm	Straight propulsion; 73 µm s^{-1} (5 wt% H_2O_2), 636 µm s^{-1} (15 wt% H_2O_2) (Table 3)	Baseline configuration
Two-orifice	1	Optical micrographs; Movie S4 (15 wt% H_2O_2)	Outer footprint as in the single-orifice baseline; two-port (chevron) inner layout (Figure 2)	Persistent curved trajectory; about 450 µm s^{-1} (15 wt% H_2O_2) (Table 3)	Broken-symmetry design
Three-orifice	1	Optical micrographs; Movies S5 (uniform 10 wt% H_2O_2) and S6 (gradient)	Outer footprint as in the baseline; three-port (three-pronged) inner layout (Figure 2)	Straight trajectory by full-clip centroid tracking; RMS lateral deviation ≈ 7% of net forward displacement (Figure S8); absolute speed not determined (no spatial calibration retained for the archived clip)	Mirror-symmetric design; gradient-biased motion response (Movie S6)
$D_{in} \approx D_{out}$ variant	1	Optical micrographs (Figure S7)	Photomask 38/40/80 µm (outer), 12/14 µm (inner) (Figure S7)	Bubble generation but no sustained forward propulsion	Control geometry ($D_{in} \approx D_{out}$)
Au-only control	1	Optical micrographs; Au-only segment of	Same outer geometry as the catalytic devices, without the Pt	No directed propulsion observed	Negative control (no Pt)

		Movie S6	catalytic surface		

Note S1. Basis for Speed Quantification in the Retained Video Clips

The absolute translational speeds reported in Table 3 were obtained from the original calibrated measurements, in which the spatial calibration was available at the time of acquisition. For the three-orifice archived clips (Movies S5 and S6), the spatial calibration was not retained, and no scale bar is embedded; therefore, absolute speed is not reported for these clips, and only the scale-independent trajectory linearity is provided (Figure S8). The basis for each clip is summarized below.

Movie	Configuration	Spatial calibration at measurement	Absolute speed reported
S1	Single-orifice (5 wt% H_2O_2)	Retained (original measurement)	73 μm s^{-1} (Table 3)
S2	Single-orifice (15 wt% H_2O_2)	Retained (original measurement)	636 μm s^{-1} (Table 3)
S3	$D_{in} \approx D_{out}$ variant (15 wt% H_2O_2)	Not applicable	No sustained propulsion
S4	Two-orifice (15 wt% H_2O_2)	Retained (original measurement)	about 450 μm s^{-1} (Table 3)
S5	Three-orifice, uniform (10 wt% H_2O_2)	Not retained	Not determined; scale-independent linearity (Figure S8)
S6	Three-orifice, gradient	Not retained	Not determined; directional response only

Supplementary Movie Descriptions

Movie S1. Single-orifice micromotor in 5 wt% H_2O_2 (73 μm s^{-1}). File: 354080-1-5%.wmv.

Movie S2. Single-orifice micromotor in 15 wt% H_2O_2 (636 μm s^{-1}). File: 354080-1-15%.wmv.

Movie S3. D_in ≈ D_out variant in 15 wt% H_2O_2 (no propulsion). File: 2016-2-17-42-15%.wmv.

Movie S4. Two-orifice micromotor in uniform 15 wt% H_2O_2 (curved trajectory, ~450 μm s^{-1}). File: media1.AVI.

Movie S5. Three-orifice micromotor in uniform 10 wt% H_2O_2 (straight-line motion). File: media2.wmv.

Movie S6. Three-orifice micromotor under an H_2O_2 concentration gradient established by an agar gel source placed to the left of the observation field. The micromotor moves in the direction opposite to the gel source (i.e., toward the low-concentration side), consistent with differential bubble generation at the lateral ports. The second half of the video shows the Au-only negative control (same three-orifice geometry, no Pt catalyst) under identical gradient conditions, exhibiting no directed motion. File: media3.wmv.
